# Role of Topical Anaesthesia in Pain Management of Farm Animals, a Changing Paradigm

**DOI:** 10.3390/ani12182459

**Published:** 2022-09-17

**Authors:** Peter Andrew Windsor

**Affiliations:** Sydney School of Veterinary Science, The University of Sydney, Camden, NSW 2570, Australia; peter.windsor@sydney.edu.au

**Keywords:** cattle, buffalo, sheep, goats, pigs, pain management, foot-and-mouth disease, vesicular virus infections

## Abstract

**Simple Summary:**

Improved on-farm animal welfare is increasingly expected by consumers of livestock products, yet motivating farmers to adopt practice changes is challenging, with many presuming increased regulations are required. Husbandry procedures are routinely conducted in livestock production globally, mostly without analgesia, despite recognition they cause pain. Similarly, debilitating transboundary viral infections, including foot-and-mouth disease (FMD), occur in numerous countries, with affected animals often treated with antimicrobial preparations and infrequently with pain relief, despite obvious suffering. A commercially available, farmer-applied spray-on topical anaesthetic formulation (TAF), containing two topical anaesthetics, an antiseptic and adrenalin in a gel matrix (Tri-Solfen^®^, Medical Ethics, Australia), provides almost instant pain relief when applied to wounds and lesions incurred during husbandry procedures and FMD infections, respectively, with field trials demonstrating that pain and suffering are markedly reduced. Additional benefits occur when the TAF is used with a non-steroidal anti-inflammatory drug (NSAID), with parenteral and oral applications increasingly available. As the TAF hastens healing rates, improves animal demeanour and provides antisepsis, the requirement for antimicrobials to manage risk of secondary bacterial infections is diminished, offering antimicrobial-resistance (AMR) stewardship. As pain management improves recovery rates, it enhances farmer animal health and welfare attitudes and increases livestock productivity and efficiency.

**Abstract:**

Field evidence indicates that livestock producers are motivated by access to products that readily deliver pain management during husbandry interventions and, more recently, viral epidermal infectious diseases, including FMD. There has been impressive adoption in Australia of a farmer-applied spray-on topical anaesthetic wound formulation (TAF; Tri-Solfen^®^, Medical Ethics, Australia), initially for managing pain of the breech modification ‘mulesing’ procedure that reduces susceptibility of sheep to flystrike. Over 120 million lambs have now received pain relief and cattle producers have commenced using the TAF for a range of husbandry procedures. This product has demonstrated efficacy for surgical castration and tail docking of lambs, surgical castration and dehorning of calves, surgical castration of piglets, debridement of lesions of the hoof for lame cattle and, importantly, treatment of clinical FMD lesions, including decubitus ulcerations occurring from prolonged recumbency. Multimodal use of an NSAID for improved pain management is advocated, particularly meloxicam, available by prescription from veterinarians for injection and as an oral formulation (Ilium Buccalgesic^®^, Troy Laboratories, Australia), with current work assessing the potential for prolonged delivery in molasses blocks. Increased use of TAF with NSAIDs significantly reduces pain and suffering in livestock, with enhanced healing of FMD lesions, reduced viral loads from Orf infections in lambs and diminished necessity of ‘antibiotic cover’, assisting antimicrobial-resistance (AMR) stewardship.

## 1. Introduction

Livestock production accounts for approximately 40% of agricultural output in developed countries [1]. The advances in animal genetics, pasture and forage improvements, superior feeding strategies, improved animal health prevention, plus other animal welfare and production management technologies have reduced land requirements for livestock by about 20%, yet doubling meat production within the last 40 years [1]. However, in subsistence livestock systems comprising smallholder farmers in developing countries, livestock production is inefficient, comprising only about 20% of agricultural output. This situation persists, despite the rapidly rising demand for milk and meat in countries that have been driving the supply of animals and products into areas where, historically, there has been limited access to proteinaceous animal-sourced foods (ASFs) [1]. 

Global meat and milk production is projected to increase another 19% and 33% by 2030, respectively [2]. Achieving this in a sustainable manner requires improved adoption of existing and emerging ‘best practice’ husbandry, welfare and climate-smart innovations, particularly as the increasing demand for ASF increases the risks of disease transmission and has potentially deleterious effects on the environment, including increased generation of greenhouse gas emissions (GHGes) [3]. Improvements are required in feed resources, preventive health strategies and biosecurity, optimal manure management, food safe processing (e.g., risk-based meat safety assurance) and both animal and product marketing. Further, provision of efficacious on-farm pain management, with rationalisation of the necessity for continuation of painful aversive husbandry interventions, is required. It is increasingly recognized that provision of pain relief for surgical husbandry interventions is urgently required. Recently, the role of pain management therapy for reducing suffering in animals afflicted by infectious disease has received attention [4]. Importantly, ruminant production is now increasingly recognised as associated with greenhouse gas emissions (GHGes). It has been estimated that improved production efficiencies could potentially assist the global livestock sector to reduce GHGes by as much as 30% [1]. However, achieving effective livestock production efficiencies requires an increased focus on improved management of the impacts of transboundary, emerging and endemic infectious diseases, with more effective strategies for managing the impacts of increasing climate variability, including preparedness for droughts, fires, storms, floods and other environmental impacts [4].

In Australia, it has been estimated that ~95% of people consider farm animal welfare as a concern, with ~91% seeking regulations ensuring transparent practices occur in livestock production [5]. Achieving this requires optimal nutritional disease prevention and welfare management. Implicit in humane animal husbandry are improved handling and transport, plus shelter for reducing the impacts of climatic extremes. Increased attention must be given to consideration of the inherent thermoneutral zones of the species and breeds of livestock that are utilised for livestock production, as this assists in managing increasing occurrences of hyperthermia and hypothermia episodes [4].

An important signpost of improving farm animal welfare has been the widespread recognition of a need to reduce and ameliorate the painful husbandry procedures that occur on farm or only conduct them with adequate pain management for reduced suffering. As pain commonly compromises animal welfare in farmed animals [6] and emerging societal and ethical concerns are demanding improvements in the welfare of animals producing food [5], there has been a considerable increase in research conducted on pain management for livestock husbandry in the past decade and a half. This includes assessments and treatments applied to the more common husbandry procedures [7] and, most recently, some diseases [4]. The increasing evidence based on pain management strategies has informed the multitudes of recommendations, extension advice, guidelines and policies that have been generated to improve animal welfare in the major farmed species, mainly in developed countries [4,6,7,8,9].

Pain is a protective biological mechanism alerting individuals to the onset of potential tissue damage and is recognised as inducing both a sensory and emotional experience that significantly affects animal welfare. When pain is unmanaged, it raises societal concerns and potentially compromises commodity markets because of deleterious impacts on the reputation and the socioeconomics of livestock production [5,9]. Pain has generally been classified as acute, inflammatory and neuropathic and although the physiological mechanisms have been reviewed [8,9], understanding of the physiology of pain perception and how to mitigate it is still developing [7]. Recent evidence indicates that pain perception pathways continue their development in the post-natal period, with events of that period impacting on subsequent pain sensitivity [7]. This supports the focus of managing painful experiences, including the husbandry interventions that are commonly afflicted upon young animals. 

Increased understanding of the mechanisms of nociception, sensitisation, cognition and modulation involved in pain expression is of relevance as it offers potential areas for enhanced amelioration of the pain experience within the pain cascade. The provision of topical anaesthesia during surgical husbandry procedures has increased the recognition of how the nociceptive pathways induce hyperalgesia and allodynia, the exaggeration or prolongation of the response to noxious inputs, versus the enabling of usually innocuous inputs to activate it, respectively [9]. Hyperalgesia occurs at the site of injury as primary hyperalgesia, with secondary hyperalgesia occurring in the surrounding adjacent and distant uninjured tissues. Hyperalgesia may be prevalent, intense, resilient and prolonged, particularly in disorders causing acute and chronic lameness in livestock [9], including foot-and-mouth disease (FMD) and sole abscesses, respectively. The management of hyperalgesia is often both complex and costly, lowering productivity, inducing deleterious behavioural, autonomic, neuroendocrine and immunologic effects, with reduced life quality that can result in mortality [9].

Whilst chronic pain in livestock is most often observed with common production diseases, including lameness, oral disorders and mastitis, the negative visual impacts of routine aversive husbandry practices have received the most attention. In Australia, this research was initially driven by a need to address the pain of the mulesing procedure, routinely conducted to prevent flystrike in some phenotypes of wool sheep. Strident welfare concerns led to studies directed at determining the efficacy of particular products designed to manage the pain occurring during mulesing, with almost immediate recognition that such products were applicable to reducing pain in other aversive husbandry procedures, including tail docking, disbudding/dehorning and castration [6,7,8,9,10,11,12,13,14,15,16,17].

There has been minimal attention, until recently, on managing the painful impacts of viral infections and other debilitating infectious disorders, causing untreated pain and suffering in livestock, particularly from the ensuing lesions of the mouth, feet and mammary tissues. Recent attention has been drawn to the pain of acute to sub-acute vesicular epidermitis that occurs when viral-induced vesicles develop and burst, causing localised ulcerative lesions that compromise prehension, locomotion and lactation from pain during FMD outbreaks [18,19]. This may be accompanied by the chronic pain and suffering caused by unresolved lesions and the deep decubitus ulcers that may develop during the prolonged recumbency occurring during recovery periods of FMD and other subacute to chronic infectious disorders. These lesions are most often treated with antimicrobial preparations. Amelioration of the pain involved has rarely received attention, unlike similar lesions in humans [15], a species fortunately able to describe pain and alert clinicians to the urgent need for pain relief.

This paper reviews aspects of ongoing advances in on-farm pain management for improved livestock welfare. It also highlights differences in awareness of pain as an issue for livestock welfare between developed and developing countries. In developed countries, delivery of pain management is increasingly expected by consumers of livestock products [5], yet routine husbandry procedures continue to be routinely conducted in many global livestock production systems, mostly without anaesthesia or analgesia. In comparison, both the concepts and practices of improving animal welfare are yet to gain traction in the smallholder livestock farming systems in many developing countries. The pain of commonly occurring infectious diseases, including FMD, remains largely unmanaged globally. Of concern is that, in such situations, there is the widespread use of often expensive antibiotics, frequently applied for a viral disease, with minimal consideration of the rapidly emerging need for improved antimicrobial-resistance (AMR) stewardship or the negative impacts on household livelihoods of such expenditure. In addition, topical applications of painful astringent preparations are common, with minimal evidence of efficacy in hastening wound healing [18,19].

In both developed and developing country scenarios, identifying motivations for farmers to adopt sustainable practice changes is challenging. Many consumers presume that increasing regulation and enforcement of compliance with standards and guidelines are required for effective animal welfare change management [4]. However, examination of recently published studies that have documented the efficacy of a commercially available, farmer-applied pain management strategy suggests an increasing willingness and capacity of producers to address pain welfare concerns in both developed and developing countries, provided products are made available that are visibly efficacious and motivate animal welfare change management. These published observations indicate that when efficacious pain management is incorporated into strategies to improve farm animal welfare, the dramatic clinical impacts observed by livestock producers applying pain relief products containing topical anaesthetics, encourages improved welfare management at the farm level and beyond. This creates a potential for increased livestock production efficiency from reduced morbidity periods. Further, the empowerment of producers to control animal pain has enabled observable increases in receptivity to adoption of other innovations to prevent or minimise disease risk, especially biosecurity and vaccination strategies. As efficacious livestock analgesia may potentially assist the change management required to address other important issues in our global food security system, including one health, ecosystem health and climate crisis concerns, this paper suggests that pain management innovations may offer a paradigm change for significantly improving livestock production efficiency.

## 2. Evaluation and Alleviation of Pain in Livestock

It is now widely accepted that animal welfare encompasses the physical health of the animals and this extends to the behavioural and emotional expression that occurs as an animal adapts to the environmental challenges of the production system. This can be particularly challenging in extensive husbandry systems where climate variability has increased the risks of environmental insults [4,20]. For many years, a useful framework to identify and describe welfare issues of farmed animals was the so-called Five Freedoms of animal welfare [20,21]. There has been increasing attention to the more recently promulgated Five Domains Model for assessing animal welfare, as the four physical domains of “nutrition”, “environment”, “health” and “behaviour”, all contribute to the fifth “mental” domain, providing an overall welfare state for the animal(s) [20,21]. Increased understanding of the broader origins of pain and how it impacts on the mental state of animals has led to the inclusion of inflammatory and neuropathic conditions amongst those entities that should receive pain management. These are potentially important developments for enabling best practice pain management and welfare advances that can create productivity efficiencies in global livestock husbandry systems.

The challenges of accurate pain evaluation in farmed animals are usually attributed to their being prey species, with the absence of overtly expressed pain or weakness tending to obscure the ready recognition and evaluation of pain [10]. Despite this, a vast array of both qualitative and quantitative techniques has now been described to assess pain. These include immediate and delayed quantification of sensory and behavioural responses, multiple and serial assessments of physiological and biochemical markers, thermal imaging and facial expression imaging, particularly as pain and suffering can induce a detectable facial ‘grimace’ score. These strategies, including a Sheep Pain Facial Expression Scale (SPFES) to identify ovine suffering, were developed and reviewed recently [4,9,10,11,12,13,14,20]. 

It has been suggested that the delayed progress in livestock pain management may be partially explained by the lack of sensitive and reliable measures of pain [8], although it has also been noted that the plethora of approaches used by different research groups has made direct and systematic comparisons between studies more difficult. There are numerous variables to consider in such comparisons, including target species, pharmaceutical agents used, age differences, dose rates, routes of administration and the combinations of painful procedures assessed (e.g., castration, with tail-docking or dehorning in sheep and cattle, respectively) that confound comparisons of published outcomes [7]. Further, whilst many alternatives for pain management therapy have been described in companion animal and human medicine, few have received much attention for use in livestock due to the complexity of livestock farming system constraints. This has correctly led to the research focus remaining largely on the use of topical and/or local anaesthetics, non-steroidal anti-inflammatory drugs (NSAIDs) or a combination of the two [4,7,8,14,16]. However, as interdisciplinary and transdisciplinary approaches between animal welfare science and human medicine may broaden perspectives on pain and provide insights and new therapies into alleviating undesirable painful states, in both livestock and humans, the ongoing dialogue between both disciplines [15] and a One Health/Welfare approach are to be encouraged in a post-pandemic world.

Of particular relevance to delays in pain welfare progress are the important constraints involved in meeting the numerous challenges of demanding livestock production systems remain focused on food safety. Developing practical, safe, affordable and cost-effective pain-management strategies that achieve rapid yet sustainable uptake by producers and can be voluntarily and repeatedly used on farm, even in periods when farm financial issues may be limiting, is both challenging and critically important. Further, they should not pose potential residue concerns and preferably contribute to improved AMR stewardship. Alleviation of pain during and after common on-farm husbandry operations involves time, cost, safety and public-health considerations [9]. This requires both the exclusion of particular drugs from administration in food producing animals (e.g., opioids) and ensuring what is available offers practicality in administration, with the exclusion of certain approaches required, including general anaesthesia, although even this has been explored in sheep [14]. For maximum uptake of pain relief for aversive procedures, including disbudding, dehorning, castration, calving and lameness, provision of visibly efficacious pain relief products that can be applied safely by farmers are required. Numerous studies have shown that topical or local anaesthesia, preferably accompanied by use of NSAID analgesia, will reduce pain during and after many of these procedures and is a robust and affordable strategy [4,7,8,10,16].

Readily available provision of effective pain management for livestock assists several important management decisions in advancing the progress of livestock production systems [4]. Firstly, it enables consideration of whether the potential benefits of an invasive husbandry procedure outweigh the deleterious effects of not incorporating the intervention in the production system. This is currently the case with the controversial mulesing procedure in flystrike-prone lines of mainly Australian Merino sheep, as the fine-wool producing phenotypes transition from dependence on the invasive procedure to genotypes offering a ‘wrinkle-free’ phenotype that is less susceptible to myiasis [12,13,14,16]. Secondly, it enables consideration of whether the immediate focus on therapy offered during a viral infectious disease outbreak should be on relief of animal suffering, rather than the more typical approach of reducing the risk of secondary bacterial infections with topical or parenteral antibiotics, risking AMR issues [14]. This is currently the case with FMD outbreaks, where both topical and parenteral antimicrobial preparations are commonly used as the preferred therapy, compromising responsible AMR stewardship, despite pain management in FMD having a dramatic impact on animal well-being and high approval rates by farmers [18,19]. Thirdly, the availability of a pain management strategy should not be permitted to compromise considerations of the epidemiological aspects of the intervention in the on-farm livestock population. The continual reassessment of the impacts of efforts to (i) reduce or remove the aversive husbandry procedure(s); (ii) address risk factors causing chronic painful disorders; or (iii) enhance the biosecurity and vaccination strategies to achieve viral disease control, should also remain as priorities for the farm manager and their advisors.

## 3. Pain Relief for Livestock Husbandry Procedures

A livestock pain relief innovation in Australia in 2005 that led to an awakening of on-farm pain welfare management, was the development of a ‘spray and stay’ topical anaesthesia and antiseptic wound formulation (TAF; Tri-Solfen**^®^**, Medical Ethics, Australia) for use during mulesing in sheep. This product, when applied to wounds, rapidly alleviates pain through blockage of nociception, minimises bleeding and provides antisepsis. With a rapid onset and prolonged duration, the readily observable clinical efficacy commenced a transformational ‘welfare revolution’ in Australian livestock agricultural attitudes and practices. On application, the TAF forms a long-lasting bio-compatible barrier over the wound, creating its own intrinsic analgesic properties and diminishing the risk of biofilm formation [15]. The TAF acts as a slow-release carrier for the actives, including the two local anaesthetics, lidocaine hydrochloride (5% *w*/*w*) and bupivacaine hydrochloride (0.5% *w*/*w*), in addition to the vasoconstrictor adrenaline acid tartrate (0.00451% *w*/*w*) and the antiseptic cetrimide (0.5% *w*/*w*). The TAF keeps the actives in contact with the wound tissue, creating synergies that prolong the analgesia well beyond the expected duration of action of actives (at least 24 h) and enhances wound healing. Adrenalin works synergistically to minimise vasodilation caused by the local anaesthetics, minimise bleeding and prevent systemic absorption and risk of toxicity [5,12,13,14,16].

The combined application of the TAF delivered directly onto the wound peri-operatively or immediately postoperatively, with or without oral or parenteral NSAIDs, was immediately recognised to have broader applications in livestock husbandry than mulesing. Numerous trials involving several routine aversive husbandry procedures have demonstrated the TAF to be safe and efficacious in managing pain and improving healing of acute surgical wounds incurred during: (i) surgical castration and tail docking of lambs; (ii) surgical castration and disbudding/dehorning of calves; and (iii) surgical castration of piglets [4,7,11,12,13,14,15,16,22,23,24,25,26,27,28,29,30,31,32,33,34,35,36,37]. The TAF has also been demonstrated to be effective in managing disorders with chronic wounds and lesions. A study in dairy cattle undergoing debridement of hoof abscess involving treatment with TAF reported significant reductions in pain during the procedure, with reduced post-surgical lameness [5,9,22]. The TAF has also been used for improving wound management in a range of situations, including: shearing cuts in sheep; lameness caused by hoof injuries and abscesses in sheep; open wounds in horses, dogs, cats and other companion animals and wildlife; and, most recently, for superficial vesicular, ulcerative and erosive lesions resulting from viral infectious diseases of the epidermis, with potential for reductions in viral loads post-therapy [18,19]. Consistent findings from these studies include regular producer confirmation of rapid onset of wound analgesia, positive welfare outcomes for an extended period well beyond that expected when the duration of action of the anaesthetic actives is considered and likelihood of improved pain management when used with an NSAID. Occasionally, there has been evidence of improved livestock productivity, mostly demonstrable in weaned animals [36].

### 3.1. Mulesing for Myiasis Management

Myiasis (flystike) is one of the most serious causes of morbidity and mortality in Australian sheep. The TAF was registered for widespread commercial use in 2012 in Australia to manage the pain and hasten the healing of open wounds incurred during mulesing in mostly Merino wool sheep that are at high risk of flystrike due to the presence of ‘breech wrinkle’, the conformation that readily retains urine and faeces and provides an attractive environment for deposition of the eggs of the sheep blowfly *Lucilia cuprina*. Following hatching, the blowfly larvae burrow into perineal tissues and the lower back, causing penetrating wounds. The afflicted animal soon becomes moribund and untreated cases usually die. Whilst long-term breeding programs for fly-resistant sheep is occurring, the mulesing procedure to create a smooth scar of unwrinkled skin of the breech and tail remnants remains the most effective method to provide life-time protection against flystrike [4,12,13,16].

The rapid adoption of TAF during mulesing performed by registered mulesing contractors and farmers in Australia has enabled the sale of wool classified as ‘PR’ (pain relief) and has improved welfare of sheep susceptible to flystrike, during the extended period required until genetic alterations in Australian Merino sheep phenotypes can progress sufficiently to successfully reduce the risk of breech myiasis. It is estimated that over 120 million lambs have now been safely treated with TAF since product registration. Importantly, plasma local anaesthetic levels have been shown to be well below toxic thresholds, even when relatively large doses were applied (up to 50 mg/kg lidocaine) in lambs. Following field reports that lambs appeared far more amenable to being moved after surgery when treated with the TAF, trials measuring wound pain and healing rates, including systematic behavioural assessments, quantitative sensory testing of wounds with von Frey monofilaments or algometry and wound photography technology established that effective wound analgesia occurred for at least 24 h, with improved wound healing [4,12,13,16].

### 3.2. Tail Docking and Castration in Lambs

Amputation of the tail in livestock or tail docking is a routine husbandry procedure in sheep production globally, for prevention of myiasis and improved fertility [15]. Tail docking causes acute pain in lambs, although is often performed without pain relief, despite increasing demands that the practice should be avoided or at least include pain management [4,14,15]. Tail docking practices vary between regions and countries, involving either rapid amputation by surgical excision or, more commonly, application of a rubber ring causing ischaemia that eventually leads to delayed removal of the necrotic tissue by sloughing. There is clear clinical evidence that the initial application of the ring causes extreme pain and close examination of animals in the period prior to sloughing of the necrotic tail remnant indicates there is also a high likelihood of pain from the chronic inflammation that occurs under and just proximal to the ring until the tissue is removed, sometimes several weeks after ring application. Estimates from the UK indicate that >90% of farmers remove tails from lambs, with rubber rings most commonly used (86%), followed by surgical (3%) and other methods (2%) [14]. Tail docking by surgery involves severing the tail using a sharp knife, scalpel, a hot knife or docking iron to cauterise the wound; it is considered a far less painful method to the use of rings on tails without application of local anaesthesia [4,7,14,15]. When surgical tail docking with and without general anaesthesia (GA) was compared, with and without inclusion of wound therapy with TAF, it was found that the TAF provided superior pain relief, improved healing, reduced wound infections, reduced cortisol responses and avoided elevation of serum amyloid A [14]. It was concluded that surgical tail docking without GA but where wounds were immediately sprayed with TAF was an affordable and more welfare-appropriate method for conducting tail docking in lambs [14].

In surgical castration of lambs, when the TAF was sprayed onto the spermatic cords and cut edges of the scrotum, lambs displayed reduced pain-related behaviour and sensory testing confirmed that hyperalgesia of the wounds was attenuated [4,7,23]. The use of local anaesthesia with NSAIDs, as a multimodal pain relief strategy for livestock undergoing routine husbandry procedures, is now recommended as best practice with recognition it provides greater amelioration of the pain response than use of a single agent alone [4,7]. Although several NSAIDs have been investigated, the use of meloxicam, available by veterinary prescription as a subcutaneous (SC) or intramuscular (IM) injection (Metacam^®^ Boehringer Ingelheim, Germany) or via oral trans mucosal (OTM) application (Ilium Buccalgesic OTM^®^, Troy Laboratories, Australia), has been most advocated. As the use of elastrator bands to cause ischaemic necrosis of the tail and scrotal tissues of lambs in Australia is favoured by many producers, an instrument to enable intravenous administration of lignocaine to the neck of the scrotum or tail, prior to application of the band(s), has also been shown to reduce pain avoidance behaviours post-procedure (Numnuts^®^, Senesino Pty Ltd., Grange, Australia) [4,7,28]. The prolonged localised inflammation within the vicinity of the elastrator ring that occurs with this technique suggests that if it is to be promulgated as a preferred option for sheep producers, then use of an NSAID should also be recommended [4,7,29].

### 3.3. Castration and Disbudding/Dehorning in Calves

In extensive cattle productive systems in Australia, most husbandry interventions, including castration and disbudding/dehorning, are performed by producers [4]. This limits the use of veterinary prescription drugs, including anaesthetics and sedatives, due to administration safety and residue considerations described above. The TAF is now increasingly a preferred pain relief product for castration on beef cattle farms, sprayed directly into the scrotum peri-operatively, with reduced postoperative pain from attenuation of hyperalgesia for at least 24 h [4,7,8]. The NSAID meloxicam is also now becoming more widely adopted by cattle producers and is available by veterinary prescription for SC or IM injection or OTM application [4,9,10], with potential for prolonged availability when consumed in medicated molasses blocks (unpublished). Meloxicam has anti-inflammatory, analgesic and anti-pyretic properties, reducing inflammation and pain-associated behaviours in castrated cattle [29]. Meloxicam is preferred over other NSAIDs for its prolonged half-life that extends duration of action to up to 72 h. The localised pain relief occurs from inhibition of cyclooxygenase (COX)-2 expression, reducing synthesis of the pro-inflammatory mediator, prostaglandin, which intensifies pain sensation and augments inflammation [30,31,32,33,34]. The use of TAF accompanied by intramuscular injections of an NSAID, administered by beef farmers under veterinary advice for disbudding and dehorning (with castration in males), appears to be rapidly increasing in northern Australian beef herd management [4]. The inclusion of an NSAID with the TAF in the pain-management protocols for this intervention is appropriate due to the risk of haemorrhage that may compromise adherence of the TAF to the disbudding/dehorning wound [34] and the potential for productivity improvements with this multimodal approach [35,36,37].

Despite the extent of duration of action of meloxicam, the pain and inflammation resulting from both surgical and ischaemic castration is prolonged, with complete healing occurring between 4 and 9 weeks post-castration [3,18,19,20,21]. As the delivery of currently available pain relief products is impractical for managing this prolonged recovery period, an alternative delivery system has been proposed, using an NSAID incorporated into a feed supplement (e.g., pellets or molasses block). The administration of NSAIDs via a feeding system has the advantage that it removes the need for animal restraint to enable administration, with the therapy consumed prior to surgical procedures then continued in the recovery period for as long as deemed necessary. The NSAID flunixin meglumine delivered via medicated pellets successfully enabled therapeutic concentrations to be established within 6 h of consumption [38]. Currently, pharmacokinetic studies (unpublished) with incorporation of meloxicam into medicated molasses blocks appear to also successfully extend the availability and duration of action of this therapeutic (D. Van Der Saag, pers. comm.) and potentially offers a convenient method of extensively prolonging pain relief post-procedure.

### 3.4. Castration in Piglets

Amelioration of the pain of castration in piglets is an important global animal welfare issue. A trial with administration of the TAF spray into castration wounds demonstrated significantly lower wound sensitivity responses for up to 4 h, compared to those castrated following intra-testicular lignocaine injection or those with no treatment [39]. A further study assessed graded nociceptive resistance movements and piglet vocal responses in addition to mechanical sensory stimulation of the wound following castration. The conclusion was that the TAF administered immediately post-skin incision followed by a minimum 30 s wait period achieved highly significant pain mitigation during castration and in the early hours following the procedure [40]. These studies demonstrate that significant pain control is achieved with the TAF during the time periods associated with maximum pain in piglets undergoing castration, providing a practical and affordable method of improving piglet welfare [39,40]. There is also potential for a multimodal approach to pain mitigation in piglets [40]. Despite these findings, challenges remain in satisfying the various and extensive animal experimentation requirements for regulatory approvals [41]. Further, uncertainties of trials meeting both the ethical obligations to minimise the number of animals needed and the agreed optimal outcome variables to be measured to validate pain mitigation, have led to continuation of delays in progressing the animal welfare gains that would follow from widespread use of this approach for piglet castration [41].

## 4. Pain Relief for Livestock Disease

### 4.1. Lameness Management in Cows

Cows with lameness due to hoof lesions often require trimming and debridement of hoof tissue. This frequently causes localised haemorrhage and exacerbates pain and distress, with animals reacting violently, endangering personnel. As pain management is rarely performed when trimming, a study assessed the efficiency of using the TAF for pain management during hoof trimming of lame dairy cows (n = 62) [9,22]. The TAF was applied immediately after the live corium was exposed, with additional applications if the animal displayed pain. Both algometry measurements performed before and after the procedure to assess hoof pressure tolerance and lameness scoring performed prior to entry and after the cow left the restraint device were conducted [9,22]. Results confirmed the TAF significantly reduced painful reactions and lameness scores during trimming and following hoof trimming, respectively, when compared with non-treated animals, with algometry values displaying increased pressure thresholds following TAF application. The study concluded that TAF improves both animal welfare and operator safety and is well suited for use by farmers due to low cost, practicality and ease of application [9,22].

### 4.2. Foot-and-Mouth Disease Therapy

**Figure 1:**
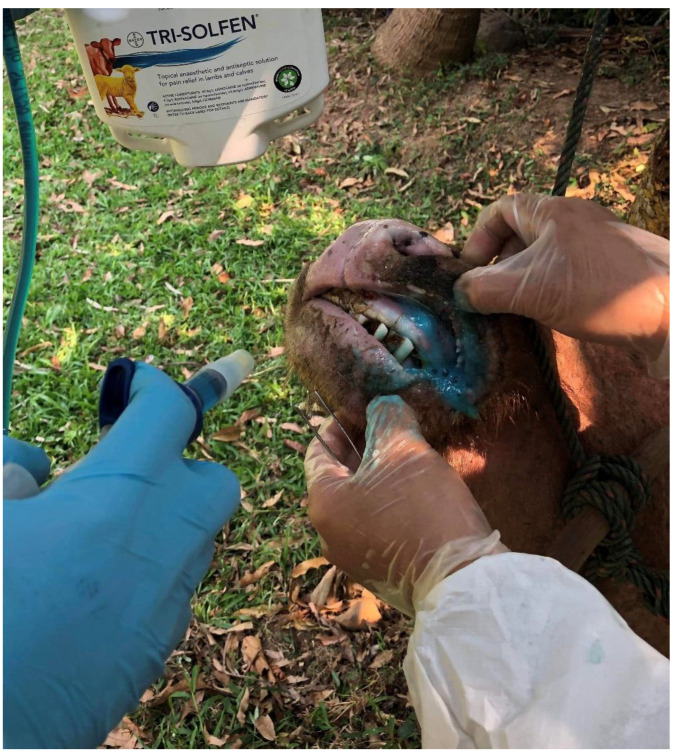
An FMD-afflicted buffalo in Lao PDR receiving oral treatment of lesions with the topical anaesthetic wound formulation (TAF), resulting in immediate improvement in demeanour.

The use of the TAF as a novel pain-relief therapeutic approach to improving livestock welfare in FMD outbreaks was conceived in 2018, following increased concerns of the widespread use of costly antimicrobial therapy for a viral disorder where secondary bacterial infections were uncommon in Lao PDR. The first trial occurred in April 2019 when the TAF product was applied to oral (Figure 1) and feet lesions of FMD in a large outbreak involving affected cattle and buffalo (n = 136) [18,42]. The immediate positive clinical impacts of spraying lesions with the TAF impressed the livestock producers and the Lao PDR animal health authorities, with local registration of the product for FMD therapy soon occurring. Then, in November 2019, a field trial comparing clinical responses and recoveries to different treatments of FMD-affected cattle was conducted during an FMD outbreak in Cameroon [19,42]. The study compared responses to treatments applied to each of three equal cohorts (n = 12), including: (i) the application of the TAF to oral and foot lesions; (ii) the administration of parenteral oxytetraycline commonly used for FMD in Cameroon and other countries; and (iii) an untreated control group. Appetite scores, lesion healing scores and changes in dimensions of lesions were recorded over a 15-day study period. Cattle treated with TAF achieved both superior appetite and lesion healing scores with more rapid reduction in dimensions of lesions than other cohorts, with a more rapid return to both eating and mobility, with earlier cessation of ptyalism (excessive salivation) and overt lameness.

**Figure 2:**
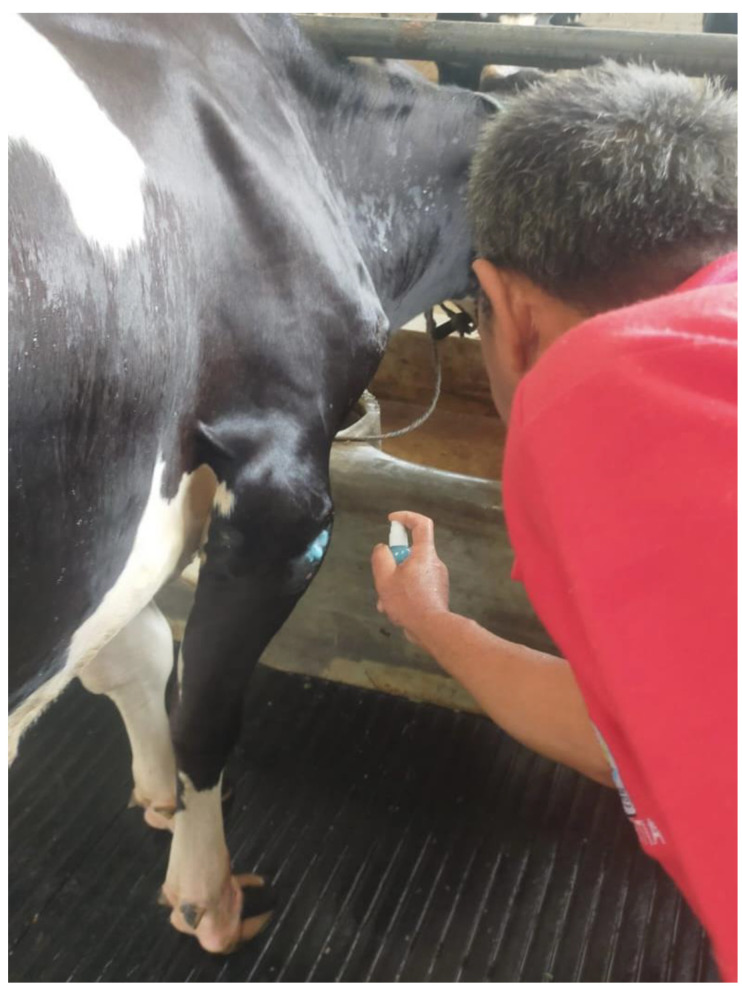
An FMD-afflicted dairy cow in Indonesia in recovery, with a chronic decubitus ulcer receiving topical spray treatment of lesions with the topical anaesthetic wound formulation (TAF) resulting in improved wound healing.

The findings from both studies confirmed that despite the low mortality rates in most FMD outbreaks, it is a debilitating and painful disease with negative animal welfare impacts that must be addressed. As farmers expressed their desire that the product be made available for use and modelling indicated that TAF therapy imposed no additional financial burden, registration of TAF for FMD in Cameroon was also achieved [19,42]. As the use of antibiotics for treatment of a viral disease potentially increases the development of AMR and residues in the food chain, it is suggested that this alternative non-antimicrobial welfare-appropriate FMD therapy should be promoted [18,19,42]. Small trials have now been conducted with TAF for FMD in several countries, including Niger, Nigeria and Kenya, with trials on both FMD lesions and the decubitus ulcers incurred from prolonged recumbency due to FMD lameness now occurring in Indonesia (Figure 2), with very promising results (unpublished observations).

### 4.3. Orf Infection

An interesting aspect of the use of the TAF for FMD, in addition to aiding healing and avoiding the need for other treatments, is that the product has a pH of ~2.7 and may offer a potential viricidal impact, possibly reducing transmission risks in disease outbreaks [18,19,42]. Although an FMD virus recovery study could not be arranged, a preliminary investigation of Orf therapy with TS examined the potential antiviral properties in the TAF in (n = 14) naturally infected lambs with Orf. The study involved viral genome real-time PCR quantification and tissue culture in ovine primary cells. Lambs were selected at the early stages of the infection and randomly divided into a cohort treated with the TAF, an untreated control group, with swans obtained prior to treatment and on days 1, 3 and 5 post-treatment. DNA was extracted with real-time PCR quantification, plus incubation with primary tissue cultures from ovine skin fibroblasts (OSF). Although no significant differences were found in the clinical progression of the lesions and PCR quantification (*p* = 0.722) between the cohorts, there was a significant difference (*p* < 0.05) in reduction in infective viral load between the groups when assessed in OSF cell cultures, suggesting that treatment of early stage lesions with TAF may reduce the infective viral load present in Orf lesions [43]. As Orf is a problematic disorder for sheep and goat exports, this therapeutic approach may have a role in assisting management of this frustratingly debilitating zoonotic disease [43,44].

### 4.4. Mycoplasma Ovis Infection

*Mycoplasma ovis* (*M. ovis, formerly E. ovis)* causes anaemia in lambs and is considered a pathogen of emerging concern, although in the Australian sheep industry, there are few reports in recent years and anecdotal observations suggest the occurrence of *M. ovis* outbreaks may possibly be declining [45]. Speculation is that this may reflect a range of factors, including a decrease in the population of Merino sheep as a proportion of the national sheep flock in recent decades, with fewer sheep requiring the mulesing operation to provide lifetime protection against myiasis, a risk factor for transmission of *M. ovis* if conducted when insects are prevalent. However, the potential decline in *M. ovis* outbreaks may also reflect improvements in hygiene and animal welfare practices during lamb marking on many sheep production enterprises in Australia [45]. Mandatory accreditation for those performing the mulesing operation, increasing use of professional contractors, declining use of blood-soaked surgical equipment, increased use of antisepsis and more appropriate administration of blowfly control products may all be of relevance to this anecdotal observation. It was also suggested that since pain welfare research commenced in 2005, the Australian sheep industry has experienced increasing uptake of the TAF for lamb marking and mulesing, with over 120 million lambs now treated. The low pH of ~2.8 and antisepsis in the TAF provides antibacterial activity that may also have contributed to reduced *M. ovis* transmission at marking, mulesing and shearing [45], although further work is required to investigate this hypothesis.

## 5. Discussion

Pain management is widely acknowledged as a priority for livestock welfare, yet progress has been described as slow compared to advances made in other species, particularly companion animals and humans [8]. This is despite the considerable research efforts to understand pain assessments and develop pain mitigation strategies for painful husbandry procedures in livestock over the past two decades, leading to the successful launch of analgesic products and protocols in a number of countries [4,7,8,9]. It is acknowledged that even with efficacious multimodal strategies, including products delivering both local anaesthetic amelioration of nociception and NSAID mitigation of sensitisation, pain is rarely obliterated or for sufficient duration. There exists a need for the eventual cessation of most aversive painful husbandry practices, when welfare management issues permit. In the interim, an improvement in available pain-mitigation strategies [7,8,9] is desirable, as is the increased adoption globally of those strategies that have become available and have been demonstrated to deliver superior welfare outcomes than the deplorable status quo.

It has been suggested as unreasonable to expect that the pain-mitigation challenge for livestock will be fully resolved in the near future [7]. This reflects the considerable constraints necessarily imposed by safe food security systems [9] that can readily lead to market failure if products continue to struggle to progress in complex regulatory approval processes, have insufficient clinical impact to be well regarded by and purchased by producers or misinformation on product risks is allowed to remain unaddressed. An example is the ongoing concerns expressed with lidocaine, with its metabolite, 2,6-xylidine, identified as a rat carcinogen [46]. This criticism persists despite lidocaine not having been associated with cancer in humans or livestock during the eight decades of therapeutic use. A recent review identified that 2,6-xylidine is a non-direct-acting (metabolic threshold-dependent) genotoxin and is not genotoxic in vivo in rats in the absence of the acute systemic toxic effects that occur at levels 35× beyond lidocaine-related exposure in humans and livestock [46]. Such challenges will likely continue to confound both the assessment of pain and evaluation of new analgesia products, particularly in commercial settings, where different measurements of pain and new drugs and strategies likely to continue to be investigated and available evidence rightly questioned. However, it is increasingly acknowledged that development of a unified measure of pain is probably inappropriate [7], particularly as livestock pain occurs in multidimensional environments and has highly variable experiential impacts and consequences. Is it also unlikely that a universal pain amelioration strategy that is suitable for use in the entirety of the highly variable livestock husbandry systems that exist globally will be appropriate or made available, particularly with the complexity of veterinary medical registration and regulatory systems that exists between countries and differences between the vast variety of global production systems (e.g., intensive/extensive, developed/developing, smallholder/corporate, etc.).

Current research, legislation and recommendations on the use of pain relief in livestock have mainly focused on the immediate response to painful husbandry procedures, particularly mulesing, castration and tail docking in sheep, castration and disbudding/dehorning in cattle and castration in piglets. Pain management for both the long-term consequences of the injuries inflicted by these aversive procedures and the acute to chronic recovery periods of common diseases involving lesions of the oral and pedal integument and mucous membranes have, until very recently, been largely neglected. This review identifies that a robust and practical multimodal strategy that is generally well regarded and self-funded by producers, as it has a broad range of applications, has now become available, particularly when the TAF is accompanied by an NSAID (e.g., meloxicam). Use of multimodal analgesia incorporating both local anaesthesia, preferably as TAF for surgical wounds or vesicular lesions to address nociception, with an NSAID to manage sensitisation, is recommended as best practice for improved livestock pain management, as it provides greater amelioration of the pain response than use of a single agent alone [4,7].

The continued systematic evaluation of multimodal approaches to the various husbandry procedure methodologies (e.g., surgical or ischaemic) and combinations of procedures (e.g., mulesing with castration) has been advised [7] and this is also relevant to pain management for both infectious epidermal diseases (e.g., FMD) and chronic neuropathic pain disorders (e.g., lameness interventions; decubital ulcer management) in livestock. Exploration of novel analgesics to prevent the development of these neuropathic changes has also been suggested as potentially providing valuable additions to a multimodal approach to management of pain associated with husbandry procedures [7], infectious epidermal diseases and chronic neuropathic disorders.

Sustained delivery of pain relief therapy is an important issue for managing the prolonged recovery periods required in both husbandry interventions and disease management. An alternative delivery system under investigation that delivers an NSAID incorporated into a feed supplement (e.g., molasses block) is particularly promising. This strategy would enable therapy to commence prior to surgical procedures and then be continued for as long as necessary, with unpublished pharmacokinetic studies, suggesting incorporation of meloxicam into highly palatable medicated molasses blocks, appears to successfully extend the availability of the pain therapeutic intervention. Molasses blocks have been advocated for delivery of improved nutrition for ruminants for many years, particularly in developing countries [47]. More recent studies in Lao PDR have identified that high-quality molasses blocks provide a robust means of delivering a multi-intervention livestock development strategy, combining nutritional and health interventions, particularly anthelmintics for endoparasite control [48,49] and urea for improved rumen fermentation [3,50]. This intervention has achieved significant improvements in livestock productivity and animal well-being. It addresses the low growth rates and declining animal weights in dry seasons that result in high greenhouse gas emissions (GHGes) per unit of meat or milk produced [3,50]. Modelling using IPCC Inventory software model V 2.69 was conducted on weight gain data from these studies (J. Hill, pers. comm.) and assessed that the likely conservative net abatement in GHGes from provision of high-quality molasses blocks to smallholder large ruminant farmers in Lao PDR was in the order of 350 kg CO_2_e over a 200-day feeding period (unpublished findings). A strategy for ‘scale-out’ to assist smallholder large ruminant livestock farming efficiency in developing countries, combining forages, health and welfare interventions and molasses blocks, has been proposed [3,50]. It offers important socioeconomic benefits for improved community resilience in poor rural communities and potentially enables reduced GHGes, by as much as 30% [2]. A recent analysis examined the proportion of published articles in livestock research (n = 563) within the period 2015–2021 that conducted animal welfare assessments that combined objective measures of physiological stress and evaluation of climate change factors in relation to livestock productivity [51]. The report identified that, although research into animal welfare assessment, objective measures of stress and climate change has been applied across both monogastric and ruminant livestock production systems, there is a shortfall of investigations on how these key factors interact to influence livestock production and the emerging technologies that can boost the quantitative evaluation of animal welfare in both intensive and extensive production systems [51]. This review aims, in part, to address that deficit.

It is important for the sustainability of livestock production that improved animal welfare occurs on all farms. The identification of efficacious and economically affordable protocols for use by farmers conducting aversive livestock husbandry procedures is deemed increasingly necessary. These protocols should be safe and, preferably, mitigate post-procedural pain after husbandry interventions at a level of sufficient impact that improved animal welfare is clearly visible to farmers. This is required to motivate their continued investment in pain management products and their acceptance that this is a necessary cost of sustainable production. The numerous studies on the use and uptake of farmer-applied, spray-on TAF for mitigation of the pain displayed by lambs, calves and piglets during and after aversive husbandry procedures suggests this is being achieved, at least in Australia [4]. Although the immediate use of topical anaesthesia after a traumatic intervention could seem contradictory, postoperative pain is more effectively addressed as the actives are rapidly delivered directly to the recently traumatised nerve fibres and tissues, with effective blockage of the nociceptors and ablation of the expected hyperalgesia. The addition of NSAIDs to the topical anaesthesia wound therapy assists in diminishing the subsequent pain escalation response pathway, improving the general welfare of the animals. In developed countries, this progress is increasingly expected by consumers of livestock products, yet routine husbandry procedures continue to be conducted in many global livestock production systems, mostly without anaesthesia or analgesia.

This review draws attention to the reality that in the smallholder livestock farming systems that mostly occur in developing countries, both the concepts and practices of improved animal welfare are yet to gain traction in many regions. In addition to husbandry interventions, the pain of common infectious diseases, including FMD, remains largely unmanaged and therapies are often inappropriate, risking AMR, financial losses and deleterious animal welfare impacts [52]. This situation exists despite numerous initiatives to address the issue, particularly through efforts driven by the Global Animal Welfare Strategy [53]. Perhaps the more recent recognition of the linkage of the Sustainable Development Goals (SDGs) and animal welfare can assist with the opinion that improving animal welfare would contribute positively to the achievement of the SDGs and, likewise, achieving the SDGs would help improve animal welfare [54]. Further, there is hopefully an awakening occurring in the post-pandemic era, that One Health is prioritised for policy development and delivery by human and animal health authorities. This may create a more receptive environment for the change management required in progressing animal health and welfare, including GHGe abatement in the broad range of livestock systems in developing countries.

## 6. Conclusions

Examination of the published studies and other reports on recent developments in pain management in livestock indicates that the application of spray-on topical anaesthesia, including the TAF (e.g., Tri-Solfen^®^), preferably accompanied by parenteral or oral administration of an NSAID (e.g., meloxicam), provides an affordable, efficacious and desired method for farmers to improve animal welfare. This protocol is appropriate for managing pain incurred during surgical husbandry procedures and is also efficacious when applied to acute to chronic disorders involving lesions of the integument, particularly those induced by some infectious viral diseases (e.g., FMD). As the TAF alleviates pain, hastens healing, reduces secondary infections and diminishes the need for unnecessary use of antibiotics that may contribute to the risks of developing AMR and residues, wider adoption of this innovation beyond the eight countries where it is currently registered is recommended. It is also suggested that as these pain-management innovations motivate practice change in developed and developing countries, they offer paradigm change for global livestock production systems that potentially assists the change management urgently required to address global food security, one health, ecosystem health and climate crisis concerns.
